# Gene Expression Patterns in Brachiopod Larvae Refute the “Brachiopod-Fold” Hypothesis

**DOI:** 10.3389/fcell.2017.00074

**Published:** 2017-08-22

**Authors:** Andreas Altenburger, Pedro Martinez, Graham E. Budd, Lars E. Holmer

**Affiliations:** ^1^Section for Evolutionary Genomics, Natural History Museum of Denmark, University of Copenhagen Copenhagen, Denmark; ^2^Department of Genetics, University of Barcelona Barcelona, Spain; ^3^Institut Català de Recerca i EstudisAvancats Barcelona, Spain; ^4^Department of Earth Sciences, Palaeobiology, Uppsala University Uppsala, Sweden

**Keywords:** Brachiopoda, body plan, evolution, brachiopod fold, gene expression, ontogeny

Brachiopods represent an animal phylum of benthic marine organisms that originated in the Cambrian. About 400 recent species are known from today's oceans (Emig et al., 2013). Around 5,000 fossil genera have been described, as brachiopods were dominant in the benthic marine environment during the Palaeozoic (Logan, 2007). Brachiopods have a biphasic life cycle with a planktonic larvae and sessile adults (Figure 1A). The phylum is divided into three clades namely Rhynchonelliformea and Craniiformea, which have short-lived lecithotrophic larvae and Linguliformea, which have long lived planktotrophic larva (Williams et al., 1996; Carlson, 2016).

**Figure 1:**
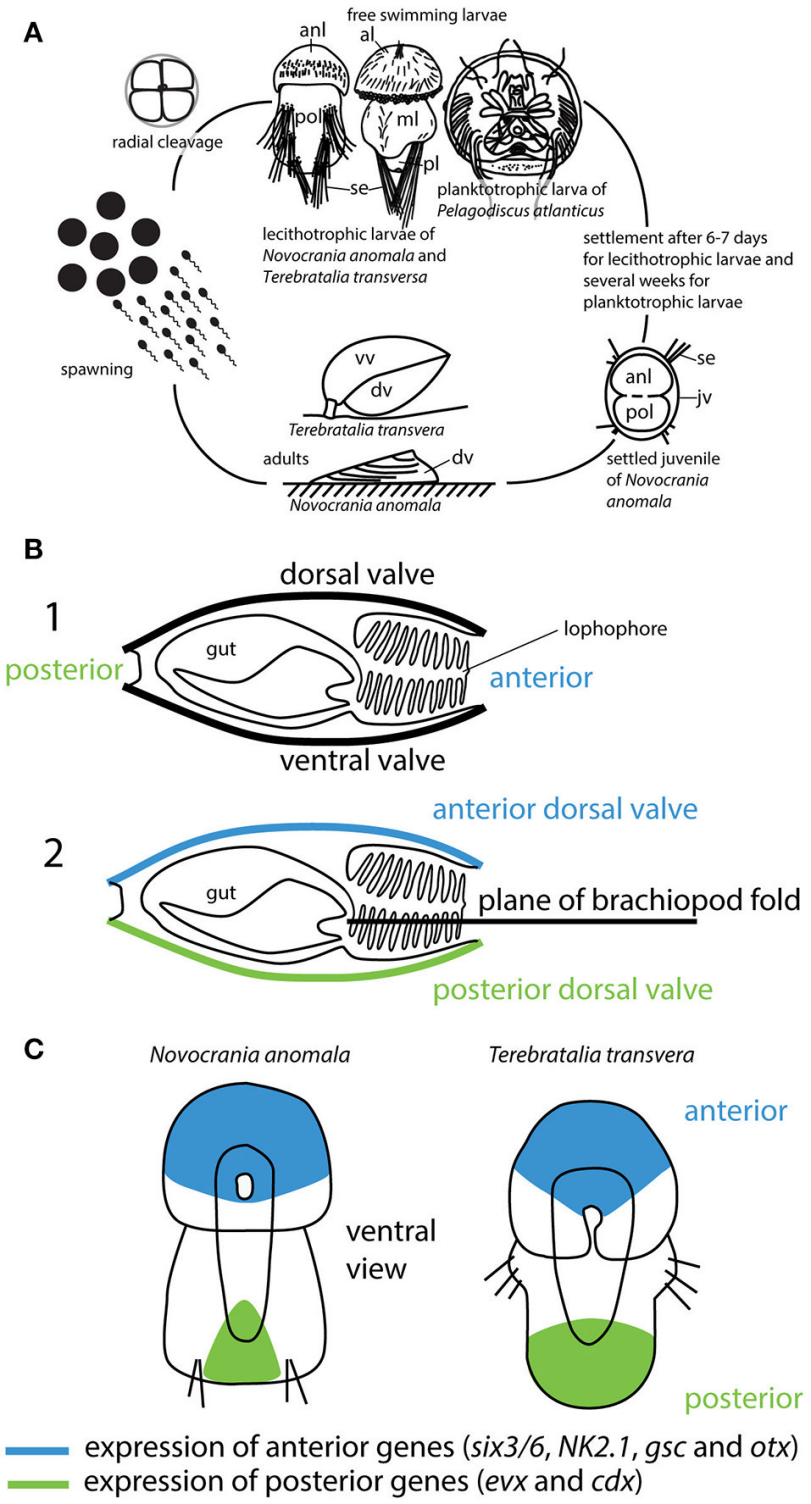
**(A)** Brachiopod lifecycle. Brachiopoda have three larval types. Rhynchonelliform larva are lecithotrophic with three larval lobes, craniiform larvae are lecithotrophic with two larval lobes, and linguliform larva are planktotrophic (for a detailed review of brachiopod development see Santagata, 2015). **(B)** Illustration of the brachiopod fold hypothesis redrawn after Cohen et al. (2003). 1. shows a hypothetical brachiopod with ventral and dorsal valve, anterior and posterior orientation. 2. According to the brachiopod fold hypothesis both valves are dorsal, one anterior and one posterior. **(C)** Gene expression patterns of “anterior” and “posterior” genes in lecithotrophic brachiopod larva redrawn after (Martín-Durán et al., 2016). Abbreviations: al, apical lobe; anl, anterior lobe; dv, dorsal valve; jv, juvenile valve; ml, mantle lobe; pl, pedicle lobe; pol, posterior lobe; se, setae; vv, ventral valve.

Although various candidate stem-group brachiopods are known (Holmer et al., 2002, 2008, 2011; Balthasar, 2004; Skovsted et al., 2009a,b), no single hypothesis of early brachiopod body plan evolution yet commands a consensus, despite the potential of the Cambrian fossil record for reconstructing early body plan evolution in this, or any other, animal phylum (Budd and Jensen, 2000; Budd and Jackson, 2016). Thus, the early evolution of brachiopods is still a matter of debate and has led to the proposal of various scenarios with varying degrees of support.

One such scenario is the hypothesis of a “brachiopod fold,” which argues that brachiopods are transversely folded across the ontogenetic anterior-posterior axis (Figure 1B) (Cohen et al., 2003; Bitner and Cohen, 2013). According to this hypothesis, both valves are considered dorsal and in order to make useful comparisons with other animal phyla along the major body axis, brachiopods should be conceptually unfolded (Cohen et al., 2003). Since its original formulation, the brachiopod-fold hypothesis has gained support by some researchers in the brachiopod community with the suggestion that brachiopods arose by the folding of a *Halkieria*-like organism containing two protective shells at either end of the body (Benton and Harper, 2009). According to the brachiopod fold hypothesis, a folding process occurs during larval metamorphosis, as a rapid muscle mediated process that moves the posterior and anterior region of the larvae close together (Cohen et al., 2003). In this context, one hint about whether or not both valves can be considered dorsal would come from the analysis of gene expression patterns of developmental genes that are highly conserved among phyla. Such genes are ancient and can be traced to the last common ancestor of bilaterian animals (Schwaiger et al., 2014). If brachiopods evolved from a *Halkieria-*like organism by folding, one would expect the expression of genes that control the anterior and posterior domains in close proximity and opposed to each other.

Several studies have investigated the expression patterns of developmental genes in lecithotrophic brachiopod larvae (Altenburger et al., 2011; Santagata et al., 2012; Passamaneck et al., 2015; Martín-Durán et al., 2016; Vellutini and Hejnol, 2016). In these analyses it has been shown that during development the genes *six3/6, NK2.1, gsc* and *otx* are expressed in the anterior domain, which becomes the apical lobe in the rhynchonelliform *Terebratalia transversa* larva, and also in the anterior domain of the craniiform *Novocrania anomala* larva (Martín-Durán et al., 2016). Conversely, the genes *evx* and *cdx* (“posterior genes”) are expressed in the area that becomes the pedicle lobe and the posterior domain of the mantle lobe in *T. transversa*, and also in the posterior domain of the posterior lobe in *N. anomala* (Figure 1C) (Altenburger et al., 2011; Martín-Durán et al., 2016). Hox genes are not expressed collinearly in these brachiopod larvae (Schiemann et al., 2017). Analysis of the Hox cluster in *T. transversa* showed a split into three subclusters similar to that observed in other spiralians, such as in the annelid *Capitella teleata* and the limpet mollusc *Lottia gigantea* (Schiemann et al., 2017). Gene expression data for individuals during metamorphosis and for juveniles are still missing. Expression of *Scr* and *Antp* in the shell-forming epithelia of *N. anomala* and *T. transversa* larva suggests a role of these genes during juvenile shell formation (Schiemann et al., 2017).

The expression patterns of “anterior” and “posterior” genes in lecithotrophic brachiopod larvae are in an anterior-posterior sequence similar to the expression domains as detected in, for example, annelids and sea urchin embryos (Wei et al., 2012; Martín-Durán et al., 2016). As the morphogenetic events occurring during metamorphosis are known for *T. transversa* and *N. anomala*, it is possible to trace the body axes to the post-metamorphic body plan, and there are no signs of a folding event. Cohen et al. (2003) based the brachiopod fold hypothesis on observations during metamorphosis of *N. anomala* (Nielsen, 1991). However, a re-evaluation of metamorphosis in *N. anomala* showed that larva settle with the posterior-most tip of the posterior larval lobe and that vental and dorsal valves are not secreted from the same tissues (Altenburger et al., 2013).

Since there is no folding event during metamorphosis in craniiform or rhynchonelliform brachiopods (Altenburger and Wanninger, 2009; Altenburger et al., 2013), we can clearly state that there is no evidence in brachiopod ontogeny that supports the brachiopod fold hypothesis. Moreover, the only known exceptionally preserved lower Cambrian rhynchonelliform brachiopod *Kutorgina chengjiangensis* clearly has a straight gut (Zhang et al., 2007), indicating that the body axis orientation of brachiopods has been retained since the Cambrian.

## Conclusion

Even though the data currently available do not allow for a conclusive hypothesis on the evolution of the brachiopod body plan, it is clear from the newly available gene expression data that the brachiopod fold hypothesis should be discarded and an alternative hypothesis for the evolution of brachiopod body plan is needed. One alternative scenario would involve a stem-group brachiopod with a tubular sclerite arrangement (Skovsted et al., 2009c; Murdock et al., 2014). A major argument for the brachiopod fold hypothesis was the presence of a U-shaped gut in some brachiopods (Cohen et al., 2003). The main group of living brachiopods which have a U-shaped gut are the Linguliformea (Kaesler, 1997; see also Carlson, 2016 for an updated phylogenetic discussion). Unfortunately, the expression patterns of “anterior” and “posterior” genes are not known for this group. This lack of data constitutes a major obstacle in trying to understand the body plan evolution within the Brachiopoda and other lophophorates. However, a U-shaped gut is already clearly present in early Cambrian stem lophophorates and brachiopods (Zhang et al., 2013, 2014), and even more recent findings (Moysiuk et al., 2017) have supported the suggestion that a tubular mode of life may be plesiomorphic within at least the lophotrochozoans (Budd and Jackson, 2016).

## Author contributions

AA designed the paper. AA, PM, GB, and LH wrote the paper.

### Conflict of interest statement

The authors declare that the research was conducted in the absence of any commercial or financial relationships that could be construed as a potential conflict of interest.
